# Endometriosis-Associated Ovarian Cancer: A Review of Pathogenesis

**DOI:** 10.3390/ijms14035367

**Published:** 2013-03-06

**Authors:** Michael J. Worley, William R. Welch, Ross S. Berkowitz, Shu-Wing Ng

**Affiliations:** 1 Division of Gynecologic Oncology, Department of Obstetrics, Gynecology and Reproductive Biology, Brigham and Women’s Hospital, Harvard Medical School, 75 Francis Street, Boston, MA 02115, USA; E-Mails: mjworley@partners.org (M.J.W.J.); ross_berkowitz@dfci.harvard.edu (R.S.B.); 2 Department of Pathology, Brigham and Women’s Hospital, Harvard Medical School, 75 Francis Street, Boston, MA 02115, USA; E-Mail: wrwelch@partners.org

**Keywords:** endometriosis, epithelial ovarian carcinoma, malignant transformation

## Abstract

Endometriosis is classically defined as the presence of endometrial glands and stroma outside of the endometrial lining and uterine musculature. With an estimated frequency of 5%–10% among women of reproductive age, endometriosis is a common gynecologic disorder. While in itself a benign lesion, endometriosis shares several characteristics with invasive cancer, has been shown to undergo malignant transformation, and has been associated with an increased risk of epithelial ovarian carcinoma (EOC). Numerous epidemiologic studies have shown an increased risk of EOC among women with endometriosis. This is particularly true for women with endometrioid and clear cell ovarian carcinoma. However, the carcinogenic pathways by which endometriosis associated ovarian carcinoma (EAOC) develops remain poorly understood. Current molecular studies have sought to link endometriosis with EAOC through pathways related to oxidative stress, inflammation and hyperestrogenism. In addition, numerous studies have sought to identify an intermediary lesion between endometriosis and EAOC that may allow for the identification of endometriosis at greatest risk for malignant transformation or for the prevention of malignant transformation of this common gynecologic disorder. The objective of the current article is to review the current data regarding the molecular events associated with EAOC development from endometriosis, with a primary focus on malignancies of the endometrioid and clear cell histologic sub-types.

## 1. Introduction

Endometriosis is a common gynecologic disorder. The estimated frequency among women of reproductive age is 5%–10% and is particularly frequent among women with pelvic pain and infertility [1–3]. This disorder is classically defined as the presence of endometrial glands and stroma outside of the endometrial lining and uterine musculature [3,4]. The exact etiology of endometriosis has yet to be elucidated. Mechanistic theories include: the reflux of endometrial tissue through the fallopian tubes at the time of menstruation, coelomic metaplasia, embryonic cell rests, and lymphatic and vascular dissemination [5–8]. However, it is generally accepted that endometriosis has a multifactorial etiology, including genetic, hormonal and immunological factors [3,6].

Areas commonly affected by endometriosis include the surface of the ovaries and the pelvic peritoneum and can result in pelvic inflammation, adhesions, chronic pain and infertility [9]. While endometriosis is a benign lesion, it shares several characteristics with invasive cancer. Similar to cancer, endometriosis has the capacity to invade and spread distantly. Endometriosis can attach to, invade and damage affected tissues [10–12]. In addition, numerous studies indicate that women with endometriosis have an increased risk of developing epithelial ovarian cancer (EOC) [11,13–18].

Utilizing the national Swedish inpatient and cancer registry database, Brinton *et al.* reviewed the records of 20,686 women hospitalized with endometriosis between 1969 and 1983. Compared to age- and period-specific incidence rates from the Swedish population at that time, this cohort had an increased overall risk of cancer, with standardized incidence ratio (SIR) of 1.2, and 95% confidence intervals (CI) between 1.1 and 1.3. The risk of ovarian cancer (SIR, 95% CI; 1.9, 1.3–2.8) was also elevated and this was particularly pronounced among women with long-standing ovarian endometriosis (SIR, 95% CI; 4.2, 2.0–7.7) [14]. A follow-up to this study was conducted to determine whether these risk ratios were maintained in an extended study with longer follow-up. Between 1969 and 2000, 64,492 women were evaluated. While the overall risk of cancer was not elevated, the elevated risk of ovarian cancer previously reported was maintained (SIR, 95% CI; 1.43, 1.19–1.71). Again, women with a long-standing history of endometriosis had an even greater risk of ovarian cancer (SIR, 95% CI; 2.23, 1.36–3.44) [15].

Ovarian cancers do not exist as a homogeneous group of malignancies, but as several histologic sub-types with unique characteristics such as frequency, disease aggressiveness, sensitivity to chemotherapy, and factors contributing to malignant development. Studies have shown that the risk associated with endometriosis varies by histologic sub-type. The greatest risk is associated with malignancies of endometrioid and clear cell histology [13,19,20]. Brinton *et al.* evaluated a population-based cohort of women in Denmark between 1978 and 1998 and determined that women with endometriosis had a predisposition to ovarian cancer. However, this association was restricted to endometrioid (relative risk (RR) of 2.53, 95% CI; 1.19–5.38) and clear cell (RR, 95% CI; 3.37, 1.24–9.14) malignancies [19]. Similarly, Rossing *et al.* interviewed 812 women diagnosed with ovarian cancer. When compared to population-based controls, the risk of endometrioid/clear cell ovarian cancer for women with endometriosis was three-fold greater. In contrast, the risk associated with other histologic sub-types of EOC was not increased [20].

While epidemiologic studies have extensively evaluated the relationship between endometriosis and ovarian cancer, the underlying mechanism and factors involved with the malignant progression of endometriosis remain poorly understood. The objective of the current article is to review the current data regarding the genetic events and molecular changes for endometriosis-associated ovarian cancer (EAOC), with a primary focus on malignancies of the endometrioid and clear cell histologic sub-types.

## 2. Malignant Transformation of Endometriosis

In 1925, Sampson was first to describe the malignant transformation of endometriosis to ovarian carcinoma [8]. It was not until 70 years later that the putative progression of endometriosis to an ovarian malignancy was supported by molecular evidence. Jiang *et al.* examined DNA from 40 cases of endometriosis to determine if these samples harbored alterations common to ovarian malignancies. Specifically, alterations in *TP53* and *RASK*, as well as allelic losses at candidate ovarian tumor suppressor loci on chromosome arms 6q, 9p, llq, l7p, 17q and 22q, were evaluated. Though no mutations were detected in *TP53* or *RASK*, loss of heterozygosity (LOH) at one or more of the evaluated loci was demonstrated in 11 of 40 cases (28%) [21]. In a similar study, Sato *et al.* evaluated the tumor suppressor gene *PTEN/MMAC1*, located on chromosome arm 10q (10q23.3) [22,23]. LOH at this site occurred in 8 of 19 ovarian endometrioid carcinomas (42.1%), 6 of 22 clear cell carcinomas (27.3%) and 13 of 23 solitary endometriotic cysts (56.5%). In addition, somatic mutations in *PTEN* were identified in 4 of 20 ovarian endometrioid carcinomas (20.0%), 2 of 24 clear cell carcinomas (8.3%), and 7 of 34 solitary endometriotic cysts (20.6%) [23].

Studies such as these support the theory that histologically benign endometriotic lesions may harbor genetic defects that are permissive for malignant transformation. This transformation is believed to progress in a step-wise fashion through an intermediary endometriotic lesion. Though the immediate precursor-lesion to EAOC has yet to be defined, several authors have evaluated potential targets that may represent the intermediary lesion. These include endometriosis contiguous with an ovarian malignancy and endometriosis displaying histologic features that distinguish it from completely benign and frankly malignant histology (*i.e*., “atypical endometriosis”). These histologic features, as defined by Czernobilsky and Morris [24] and LaGrenade and Silverberg [25], include large hyperchromatic or pale nuclei with moderate to marked pleomorphism; an increased nuclear-to-cytoplasmic ratio; and cellular crowding, stratification, or tufting. According to a study by Fukunaga *et al.*, such atypical endometriosis was identified in 54% of clear cell and 42% of endometrioid ovarian carcinomas [26]. Separately, Ogawa *et al*. determined that in 37 cases of ovarian carcinomas with endometriosis, 22 cases showed transition from typical endometriosis to atypical endometriosis, and 23 cases showed transition from atypical endometriosis to carcinoma [17]. Ballouk *et al*. found that half of the endometriotic cysts with severe atypia showed DNA aneuploidy, suggesting the potential of invasive epithelial malignancies arise in these atypical endometriotic cysts [27].

In the previously mentioned study by Sato *et al.*, synchronous endometriosis was identified in five cases of endometrioid and seven cases of clear cell carcinoma. In the five endometrioid carcinomas synchronous with endometriosis, three displayed LOH events common to both the carcinoma and the endometriosis. Among the seven clear cell carcinomas synchronous with endometriosis, three displayed LOH events common to both the carcinoma and the endometriosis [23]. Protein markers specific for ovarian malignancies have also been evaluated in the context of contiguous or atypical endometriosis. Hepatocyte nuclear factor-1β (HNF-1β) is a transcription factor that has been shown to be significantly up-regulated in ovarian clear cell carcinoma and rarely expressed in non-clear cell carcinoma specimens [28]. Evaluating HNF-1β, Kato *et al.* identified 17 clear cell tumors associated with endometriosis. Endometriotic epithelium was identified in 12 of these 17 cases, with a total of nine displaying expression of HNF-1β. Though histologically benign, the nine cases of endometriosis that displayed HNF-1β expression included five cases of reactive epithelium and four cases of “atypical endometriosis.” When a series of 40 cases of endometriosis not associated with a primary clear cell ovarian carcinoma were also evaluated, 16 (40%) of the cases displayed HNF-1β positivity. However, the authors noted that expression of HNF-1β was almost exclusively detected in the epithelium showing inflammatory atypia. Kato *et al.* thus concluded that some epithelial cells of ovarian endometriosis, including atypical endometriosis and endometriosis that undergoes repeated regenerative and inflammatory changes, have already acquired a clear cell phenotype [29].

Most recently, by transcriptomic sequencing, Wiegend *et al.* identified mutations of the tumor suppressor gene *ARID1A* that are common to endometrioid and clear cell ovarian carcinomas. Of 119 ovarian clear cell carcinomas and 33 endometrioid carcinomas, *ARID1A* mutations were present in 55 (46%) and 10 (30%) cases, respectively. Two sets of patient samples containing a clear cell carcinoma associated with contiguous atypical endometriosis were further evaluated. *ARID1A* mutations were found in the primary malignant lesion and the contiguous, atypical endometriosis. *ARIDA* expression was retained, however, in areas of endometriosis from sites distant from the primary malignant lesion [30]. In an immunohistochemical evaluation of ARID1A protein expression in 28 cases of endometriosis-associated clear cell carcinomas, Yamamoto *et al.* found that 17 cases (61%) were ARID1A negative. Within these cases, all atypical endometriosis and 86% of non-atypical endometriosis also lost ARID1A expression, suggesting that loss of ARID1A expression occurs as a very early event in the malignancy development [31].

## 3. Pathways of Carcinogenesis

Recent whole genome or targeted sequencing studies have identified frequent mutations of *ARID1A* and *PIK3CA* genes, and moderate mutations of *PPP2R1A* and *KRAS* in ovarian clear cell carcinomas [30,32,33], mutations of *PTEN*, *CTNNB1* and *KRAS* in endometrioid cancer [34,35]. In combination with the results of gene expression profiling of these two tumor types [36–39], activation of oncogenic KRAS and PI3K survival pathways, and inactivation of tumor suppressor genes *PTEN* and *ARID1A* are suggested for clear cell and endometrioid ovarian carcinomas, respectively. We emphasize here the significance of the following pathways in clear cell and endometrioid tumors in association with endometriosis.

### 3.1. Oxidative Stress

Oxidative stress has been implicated in the development of numerous human disorders. These include cancer, atherosclerosis, diabetes, cardiovascular disease, neurodegenerative diseases, pulmonary fibrosis, liver diseases, AIDS and aging [40]. As mentioned previously, endometriosis is defined as the occurrence of endometrial tissue outside of the uterus, often on the surface of the ovaries and the pelvic peritoneum [9]. Repetitive hemorrhage and the accumulation of heme and free iron within endometriotic lesions are believed to play a pivotal role in the development of ovarian carcinoma through the formation of reactive oxygen species (ROS) [41–43]. Yamaguchi *et al.* evaluated the contents of 21 endometriotic cysts, 4 clear cell carcinomas, and 11 non-endometriotic cysts to determine the concentrations of free iron and several stress-related factors (e.g., lactose dehydrogenase, potential antioxidant lipid peroxide, and 8-hydroxy-2′-deoxyguanosine). When compared to non-endometriotic cysts, the concentration of free iron in endometriotic cysts was significantly higher (*p* < 0.01). In addition, the average concentration of the evaluated stress-related factors was significantly greater in endometriotic cysts, when compared to non-endometriotic cysts. *In vitro* analyses showed that the contents of endometriotic cyst could produce more ROS and could induce gene mutations more frequently than the contents in the other cysts [42]. Similarly, Sanchez *et al.* found that markers of oxidative damage, such as strand breakage, DNA adducts and lipid peroxidation products could all be detected in ovarian cancer tissue [44].

Ultimately, oxidative stress results when the production of ROS exceeds the capacity of cellular antioxidant defenses to remove toxic ROS [45,46]. Excess ROS can result in oxidative stress-related damage to proteins, lipids, DNA and the cell membrane through numerous carcinogenic DNA mutations or losses [47,48]. The theory that oxidative stress is implicated in the development of ovarian carcinoma, specifically clear cell carcinoma, is further supported by microarray gene-expression profiling [29,49]. Fifty-four genes have been found to be highly up-regulated in ovarian clear cell carcinoma. Of these 54 genes, 47 (87%) are redox-related genes, including oxidative and detoxification enzymes [43]. The subsequent carcinogenic pathways remain poorly understood but HNF-1β appears to play a key role as 22 (40.7%) of the 54 up-regulated genes are downstream targets of HNF-1β [49–51].

HNF-1β is a transcription factor that targets genes encoding proteins vital to cell proliferation, differentiation, gluconeogenesis and glycogen synthesis [52]. The relationship between HNF-1β and oxidative stress has been studied in the context of viral hepatitis infection. Hepatitis C virus (HCV) infection induces oxidative stress during replication and/or protein expression, and chronic HCV infection in human liver, as well as in different experimental models, is associated with excess oxidative stress [53–55]. Adaptive responses to the increased oxidative burden include the activation of detoxification pathways via activation of HNF [55–57]. With respect to ovarian carcinoma, the oxidative stress related HNF-1β pathways remain largely unexplored. However, an interesting study conducted by Liu *et al.* revealed that down-regulation of HNF-1β by shRNA strategy sensitized ovarian cancer cells to cisplatin- or paclitaxel-induced cytotoxicity both *in vitro* and *in vivo*[58].

### 3.2. Inflammation

In 1863, Rudolf Virchow observed leukocytes in neoplastic tissues and made a connection between inflammation and cancer. He suggested that the “lymphoreticular infiltrate” reflected the origin of cancer at sites of chronic inflammation [46]. Both acute and chronic inflammations are classic features of endometriosis and a growing body of literature now supports the conceptualization of endometriosis as a pelvic inflammatory condition [59,60]. In women with endometriosis, the peritoneal fluid often contains an increased number of activated macrophages [61]. In addition, several important cytokines and chemokines are present in increased concentrations. These include TNF-α, IL-1β, IL-6, IL-8, regulated on activation normal T cell expressed and secreted (RANTES), and monocyte chemotactic protein-1. The latter three are chemoattractants, which facilitate the recruitment of macrophages [62–65].

Similar to results in endometriosis, numerous studies suggest inflammatory mediators and several cytokines (e.g., TNF-α, IL-1β, IL-6) promote the development, growth and progression of EOC [66,67]. Szlosarek *et al.* explored the role of TNF-α in the development of ovarian carcinoma. Within a cancer profile array including serous and clear cell ovarian carcinoma, the expression of TNF-α was increased when compared to the level of expression within normal ovarian tissue. Further, cultured ovarian cancer cells expressed up to 1000 times more TNF-α mRNA than cultured normal ovarian surface epithelium [68]. A follow-up study from the same group showed that elevated TNF network gene expression in the tumor microenvironment resulted in increased signaling related to angiogenesis, cell adhesion, cell cycle, inflammation, as well as NOTCH signaling [69]. In another microarray expression profiling study of endometriosis-associated endometrioid ovarian cancer, the expression of small inducible cytokine A2 (SICA2) and small inducible cytokine subfamily A, member 14 (CCL14) were the most up-regulated genes in endometriosis-associated endometrioid ovarian tumors when compared with benign ovaries and endometrioid cases not associated with endometriosis, suggesting that inflammatory cytokines are important in the etiology of endometriosis and associating endometrioid tumors [36]. Finally, prostaglandin E_2_ (PGE_2_) is a central mediator of the inflammatory response but has also been shown to regulate vital processes related to tumor growth, including angiogenesis, proliferation and inhibition of apoptosis [70,71].

### 3.3. Estrogen

Numerous studies have demonstrated an association between hyperestrogenism and gynecologic malignancies. These include cancers of the breast, endometrium and ovary [72–74]. Specific to EAOC, hyperestrogenism has been associated with the malignant transformation of endometriotic cysts and the micro-environment provided by endometriosis facilitates the accumulation of excess estrogen through several mechanisms [75,76]. For example, the enzyme aromatase is normally absent in eutopic endometrial tissue, but present in high levels within endometriotic tissue [77]. This enzyme catalyzes the conversion of androstenedione and testosterone, derived from ovarian and adrenal sources, to estrone and estradiol (E_2_), respectively [78]. In endometriotic tissue, this results in the constitutive expression of E_2_[79]. Next, endometriotic tissues lack the enzyme 17β-hydroxysteroid-dehydrogenase (17β-HSD) type 2, which is present in eutopic endometrial tissue and serves to convert E_2_ to estrone, which is a less potent estrogen [80]. In contrast, 17β-HSD type 1 converts estrone to the more potent E_2_ and this enzyme can be found in endometriotic tissue with the cumulative effect of increasing the local concentration of highly estrogenic E_2_ through increased production and decreased inactivation [78,80].

Excess E_2_ can result in cellular proliferation through the stimulation of cytokine production, specifically IL-8 and RANTES [81]. In addition, E_2_ stimulates the production of PGE_2_. As previously mentioned, PGE_2_ promotes tumor growth, but also serves to stimulate the activity of aromatase, thereby resulting in a positive feedback loop in favor of continuous estrogen formation in endometriosis [77,78]. In sum, the micro-environment in endometriotic tissue is marked by proliferative pressure with an enhanced level of reparative activity and thus, a higher chance for DNA damage and mutations.

Among EAOC, expression of estrogen receptor (ER) and progesterone receptor (PR) is predominantly found in the endometrioid sub-type. The clear cell sub-type typically does not express ER or PR [82]. The previously described events, mediated by oxidative stress and inflammation secondary to the repeated hemorrhage from endometriosis, result in DNA methylation that has been linked to decreased ER expression [83,84]. Some investigators have postulated a dualistic model for the development of EAOC, in which the loss of ER expression marks a pivotal point in the carcinogenic pathway separating the development of estrogen-dependent carcinomas (*i.e*., endometrioid) from estrogen-independent carcinomas (*i.e*., clear cell) [85,86]. While the separation of estrogen-dependent and estrogen-independent EAOC is supported on the basis of morphology and genomics, the details of both pathways remain areas of further investigation [85,86].

## 4. Conclusions

The association between endometriosis and EOC has been supported by years of epidemiologic research. Our current knowledge of this association at the molecular level remains an area of great interest and worthy of future studies as the carcinogenic mechanisms and pathways remain poorly defined. Future studies will clarify the roles of oxidative stress, inflammation and estrogen in the development of EAOC. At this point, we know that the micro-environment of endometriosis and EAOC share similar cytokines and mediators. Whether this similarity represents a link between endometriosis and EAOC or simply an employment of common signaling molecules to two separate lesions remains to be seen. Studies evaluating the possible intermediary lesion of EAOC (both contiguous and “atypical” endometriosis) appear promising in strengthening the link between endometriosis and ovarian carcinoma. These studies may also aid in identifying endometriotic lesions at greatest risk of malignant transformation. However, most of the specimens harboring these features are in formalin-fixed, paraffin-embedded (FFPE) form, which presents a challenge for successful molecular profiling and analysis. In conclusion, EAOC represents a poorly understood consequence of a common gynecologic disorder (*i.e*., endometriosis). A greater understanding of the pathogenesis of EAOC would potentially allow for preventative strategies for those women with endometriosis at greatest risk of developing EAOC, as well as novel treatment approaches for women already diagnosed with EAOC.
